# Methotrexate-Associated Pneumonitis and Rheumatoid Arthritis-Interstitial Lung Disease: Current Concepts for the Diagnosis and Treatment

**DOI:** 10.3389/fmed.2019.00238

**Published:** 2019-10-23

**Authors:** George E. Fragoulis, Elena Nikiphorou, Jörg Larsen, Peter Korsten, Richard Conway

**Affiliations:** ^1^First Department of Propaedeutic Internal Medicine, National and Kapodistrian University of Athens, Laiko General Hospital, Athens, Greece; ^2^Institute of Infection, Immunity and Inflammation, University of Glasgow, Glasgow, United Kingdom; ^3^Department of Inflammation Biology, Faculty of Life Sciences & Medicine, Centre for Rheumatic Diseases, School of Immunology and Microbial Sciences, King's College London, London, United Kingdom; ^4^Department of Diagnostic and Interventional Radiology, University Medical Center Göttingen, Göttingen, Germany; ^5^Department of Nephrology and Rheumatology, University Medical Center Göttingen, Göttingen, Germany; ^6^Department of Rheumatology, Blackrock Clinic, Dublin, Ireland

**Keywords:** rheumatoid arthritis, interstitial lung disease, methotrexate, biologics, immunosuppressive therapies

## Abstract

Rheumatoid arthritis (RA) is a type of inflammatory arthritis that affects ~1% of the general population. Although arthritis is the cardinal symptom, many extra-articular manifestations can occur. Lung involvement and particularly interstitial lung disease (ILD) is among the most common. Although ILD can occur as part of the natural history of RA (RA-ILD), pulmonary fibrosis has been also linked with methotrexate (MTX); a condition also known as MTX-pneumonitis (M-pneu). This review aims to discuss epidemiological, diagnostic, imaging and histopathological features, risk factors, and treatment options in RA-ILD and M-pneu. M-pneu, usually has an acute/subacute course characterized by cough, dyspnea and fever. Several risk factors, including genetic and environmental factors have been suggested, but none have been validated. The diagnosis is based on clinical and radiologic findings which are mostly consistent with non-specific interstitial pneumonia (NSIP), more so than bronchiolitis obliterans organizing pneumonia (BOOP). Histological findings include interstitial infiltrates by lymphocytes, histiocytes, and eosinophils with or without non-caseating granulomas. Treatment requires immediate cessation of MTX and commencement of glucocorticoids. RA-ILD shares the same symptomatology with M-pneu. However, it usually has a more chronic course. RA-ILD occurs in about 3–5% of RA patients, although this percentage is significantly increased when radiologic criteria are used. Usual interstitial pneumonia (UIP) and NSIP are the most common radiologic patterns. Several risk factors have been identified for RA-ILD including smoking, male gender, and positivity for anti-citrullinated peptide antibodies and rheumatoid factor. Diagnosis is based on clinical and radiologic findings while pulmonary function tests may demonstrate a restrictive pattern. Although no clear guidelines exist for RA-ILD treatment, glucocorticoids and conventional disease modifying antirheumatic drugs (DMARDs) like MTX or leflunomide, as well as treatment with biologic DMARDs can be effective. There is limited evidence that rituximab, abatacept, and tocilizumab are better options compared to TNF-inhibitors.

## Introduction

Rheumatoid arthritis (RA) is the most common inflammatory arthritis with a worldwide prevalence of about 1% and a female predominance of about 3:1 (1). While there are numerous synthetic and biologic disease-modifying antirheumatic drugs (DMARDs) that can halt progression of the articular manifestations of the disease, data on extraarticular manifestations are less conclusive. Over the past few years, the lung has become a major focus in terms of pathophysiology and overall prognosis (2). In clinical practice, there are perceived discrepancies regarding pulmonary toxicity between pulmonologists and rheumatologists, especially regarding methotrexate (MTX) and the potential risks of long-term pulmonary fibrosis. Over the past few years, more evidence has evolved adding to the controversy. To make matters more complex, the pulmonary toxicity of biological therapies is less clear. Therefore, rheumatologists are frequently faced with the situation of how to treat joint manifestations effectively in the presence of interstitial lung disease (ILD) since evidence regarding pulmonary safety is sparse. In this review article, we aim to summarize the available evidence regarding MTX-associated pneumonitis (M-pneu), RA-ILD, and discuss treatment options based on available evidence.

## Methods and Literature Selection

A focused literature review including the keywords “methotrexate,” “pneumonitis,” “interstitial lung disease,” and “rheumatoid arthritis” was performed. In addition, articles from the personal archives of the authors or references from key papers were included if deemed relevant by the authors.

## Pulmonary Disease Patterns in Rheumatoid Arthritis

### Methotrexate-Associated Pneumonitis

#### Epidemiology

The frequency of M-pneu has been reported to range between 0.3 and 11.6% (3–6), depending on the methodology used and the criteria applied for M-pneu diagnosis. Interestingly, since 2001, no cases of M-pneu have been reported in randomized clinical trials of MTX in RA (7). M-pneu generally has an acute or subacute course and is usually observed within the first year of treatment (8). However, cases of late-onset M-pneu have been also described (9, 10).

#### Clinical Symptomatology and Laboratory Findings

Symptomatology mainly pertains to dry cough and dyspnea observed in more than 80% of the patients. Fever also occurs in more than 60% of them (3, 11, 12). Some authors have suggested that mild peripheral blood eosinophilia is present in about 25–40% of patients with sub-acute M-pneu (4, 9–11). Also, in case-series from patients with M-pneu it was demonstrated that peripheral blood lymphocytes dropped at the time of M-pneu and went back to normal after recovery (13). These findings, although very useful in everyday clinical practice, remain to be confirmed in larger studies.

#### Pathogenesis and Risk Factors for the Development of M-Pneu

Pathogenic mechanisms underlying M-pneu are unclear. It is considered by many investigators to be a hypersensitivity reaction, while interleukin-8 has been implicated in the pathogenesis (14). It should also be noted that patients receiving MTX are also at an increased risk for developing MTX-related lymphoproliferative disorder (LPD) (15). Interestingly, LPD regresses in many cases after the withdrawal of MTX (15, 16). Recent studies investigating the clinical and histopathologic characteristics of these patients have shown that in half of these cases this is linked to Epstein-Barr virus infection (15, 17) with p38 MAP kinase, PI3 kinase, and MEK pathways being implicated (18). The lung can also be involved in the context of MTX-related LPD (15, 16, 19, 20): Cases of lung lymphomatoid granulomatosis, a rare entity characterized histologically by multiple nodular lesions and vessel wall infiltration by lymphoid cells, have been described (16, 19, 20).

Several risk factors have been identified (Table 1), but it is remains uncertain to what extent they contribute to the occurrence of M-pneu. These factors include: age more than 60 years, diabetes mellitus, hypoalbuminemia, previous use of DMARDs), renal dysfunction, male gender, increased Health Assessment Questionnaire (HAQ) score, decreased pain Visual Analog Scale (VAS) score and pre-existing lung disease (6, 12, 21–23). However, these have not been replicated in other studies (24). Genetic factors might also play a role. In a Japanese population, an association between M-pneu and the HLA-A31:01 haplotype has been described (25). However, in a Genome Wide Association Study in a United Kingdom population, these results were not reproduced, but three Single Nucleotide Polymorphisms (SNPs) have been found to be associated with M-pneu occurrence with borderline significance (26). Environmental factors also possibly contribute. It has been suggested that increased latitude is related to an increased risk for M-pneu development. In fact, Jordan et al. using data from the New Zealand ministry of health showed that the incidence rate ratio for M-pneu was increased by 16% per one degree of increasing latitude (27).

**Table 1:** Proposed risk factors for the development of methotrexate-associated pneumonitis (M-pneu) and rheumatoid-arthritis-interstitial lung disease (RA-ILD).

**Risk factors**	
**M-pneu**	**RA-ILD**
Pre-existing lung disease	Disease activity
Age > 60 years	Age
Male sex	Male gender
Diabetes mellitus	Smoking
High HAQ score, low pain VAS score	Positive rheumatoid factor
Chronic kidney disease	Positive anti-citrullinated peptide antibody
Hypoalbuminemia	MUC5B promoter variant rs35705950
Previous use of DMARDs	
Genetic factors (e.g., HLA-A31:01)^*^	
Environmental factors (e.g., latitude)	

*DMARDs, disease-modifying antirheumatic drugs; HAQ, health assessment questionnaire; HLA, human leukocyte antigen; VAS, visual analog scale*.

* *Not confirmed in all populations*.

#### Diagnosis

A diagnosis of M-pneu is based on the clinical and radiologic findings. Other diagnostic modalities like pulmonary function tests (PFTs) and bronchoalveolar lavage (BAL) might prove to be helpful as well. However, the differential diagnosis, which includes infections, like *Pneumocystis jirovecii* pneumonia (PJP), viral and atypical pneumonias, and ILD due to RA (RA-ILD), is difficult to be made (11).

Performance of PFTs routinely for diagnostic or prognostic purposes is still under debate (12). Although some studies have demonstrated only a minor effect of MTX on PFTs (28), two prospective studies have found that there are some alterations: Khadadah et al. (29), describe that after 2 years of treatment of low-dose MTX, patients may develop a restrictive pattern with significant decline in total lung capacity (TLC), functional residual capacity (FRC), forced expiratory volume in 1 s (FEV1), forced vital capacity (FVC), and an increase in the FEV1/FVC ratio. Similarly, Cottin et al. (30), examining 124 patients treated with MTX, described a reduction of FVC, FEV1, and diffusing capacity of the lung for carbon monoxide (DLCO)/alveolar volume (VA). However, these changes could not predict the 3.2% of patients who developed M-pneu in their study (30). On the other hand, Saravanan et al. (8), have suggested that PFT abnormalities [low FEV1, vital capacity (VC) and diffusing transfer of the lung for carbon monoxide (TLCO)] might have a prognostic role, carrying a higher risk for M-pneu development in RA patients.

Of note, in published guidelines for MTX treatment in RA, based on literature review and expert opinion it is stated that PFTs with DLCO should be performed in patients with pre-existing lung disease or current symptoms (low strength of recommendation [D]) (6). In pediatric populations, some studies do not describe any abnormalities in children with juvenile idiopathic arthritis (JIA) treated with MTX (31, 32), while others conclude that there are some alterations in PFTs, like decrease of the mid-mean expiratory flow (MMEF) and DLCO (33, 34) or an increase in the TLC, FRC and residual volume (RV) (35). However, these are not affected by MTX and they were rather attributed to JIA *per se*. Besides, none of these patients developed clinically significant lung disease in these studies (33).

BAL examination is often performed in these patients. Most investigators agree that a lymphocytic pattern is observed (36), although cases of with BAL neutrophilia have been also reported (10, 37). Lymphocytosis in BAL is not specific for M-pneu as it is also seen in interstitial pneumonitis due to RA (36, 38) and in RA patients treated with MTX without respiratory symptoms (39). A recent systematic literature review examining characteristics of BAL in M-pneu has shown that lymphocytosis was present in the majority (89%) of BAL samples, while high levels of neutrophils were present in only 17% (40). In fact, six cytological patterns were identified (four with predominant lymphocytosis and two in which neutrophilia was the principal finding (40). It has been also suggested that predominance of CD4^+^ T cells in BAL is suggestive of M-pneu (36) but there is some evidence that an increased CD4/CD8 ratio can also be found in other RA patients, usually those with pulmonary involvement (40). Also, the CD4/CD8 ratio can be found low or normal in about half of the M-pneu patients. Chikura et al. suggested that neutrophils are increased in the BAL of patients with M-pneu having received treatment for <6 months and with a cumulative dose of <300 mg, while the opposite was the case for lymphocyte numbers (41). These results were independent of the indication for which MTX was given (i.e., RA, Primary biliary cholangitis, Psoriatic arthritis, and others). Finally, serum levels of KL-6, a glycoprotein antigen, and surfactant protein D, both expressed mainly by type II pneumocytes, have been proposed as biomarkers for diagnosing and monitoring M-pneu (42). However, they are found to be increased in other lung diseases as well (43), therefore their utility, if any, in the setting of M-pneu remains to be defined.

Transbronchial lung biopsy (TBLB) might also be a useful diagnostic adjunct. In a study evaluating 44 patients with drug-induced lung injury, 75% underwent TBLB (44). TBLB was diagnostically helpful in 75%. Although histopathology alone cannot diagnose M-pneu, it may provide useful supplemental information that can be incorporated with clinical, radiologic, laboratory, and other features in the final diagnosis (44).

#### Imaging Features

**Figure 1:**
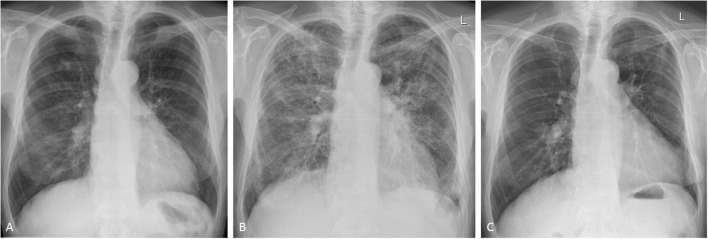
Methotrexate-induced pneumonitis in a 77-year-old man with rheumatoid arthritis. **(A)** Posterior-anterior chest radiograph immediately before the initiation of treatment. Following 10 days of methotrexate, the patient experienced progressive dyspnea and fever. Follow-up chest radiography showed bilateral heterogeneous opacities in all lung zones. **(B)** The patient was transferred to the intensive care unit for supportive treatment. High-dose glucocorticoids were administered and gradually withdrawn following clinical and radiological improvement. Initial high-resolution CT scanning showed diffuse infiltrates and bilateral patchy consolidations with only very limited ground-glass opacities (images not shown). **(C)** Seven months after stopping methotrexate, the changes of pulmonary toxicity had fully resolved.

Radiological findings reflect the underlying histopathologic process and include mostly non-specific interstitial pneumonia (NSIP), more so than bronchiolitis obliterans organizing pneumonia (BOOP) (45): on chest radiography, M-pneu gives rise to diffuse heterogeneous opacities in NSIP or bilateral scattered heterogeneous or homogeneous opacities with a peripheral distribution in the upper and lower lobes in BOOP. On CT scanning, scattered or diffuse ground-glass opacities are seen in early NSIP and basal fibrosis in the later stages of the disease. In BOOP, poorly defined nodular consolidations, centrilobular nodules, bronchiolitic (tree-in-bud) changes and bronchial dilatation are the dominant features (Figures 1A–C) (6, 46). In a study examining CT findings in M-pneu, it was found that in the majority of the patients, these lesions subsided during a mean follow-up period of 31 days (46).

#### Histologic Findings

The most common histopathological pattern observed includes interstitial infiltrates by lymphocytes, histiocytes, and eosinophils with or without granulomas (36). Granulomas, usually non-caseating, are also identified in some patients, while hyperplastic type II pneumocytes and perivascular inflammation are also commonly seen (47). Other patterns have also been described and often coexist with interstitial pneumonitis, such as diffuse and organized alveolar damage (3, 12, 47). The latter seems to be more frequent in acute cases of M-pneu (47).

#### Treatment

In suspected M-pneu MTX should be discontinued immediately. Often, treatment with steroids is required (8). Other immunosuppressive drugs, such as cyclophosphamide (CYC), have also been administered successfully (48). Tocilizumab (TCZ), given its efficacy as monotherapy in RA, is also an attractive therapeutic option, since its use has been reported to be beneficial (38).

#### Prognosis

The prognosis of M-pneu is generally good and most patients recover fully (8), however, mortality is reported to be relatively high reaching 17.6% (6, 11). Other smaller studies have reported even higher figures up to 30% (49). Besides, in a review assessing patients (including individuals with RA) who developed M-pneu, the percentage was 13% (47). Furthermore, a study by Chikura et al. examining 56 RA patients with M-pneu suggested that mortality was more increased in patients who developed pneumonitis after treated with MTX for <6 months compared to those treated for a longer time period (41). It is suggested that this difference in mortality is accompanied by specific histopathologic features and characteristics in the BAL examination (41). Re-introduction of MTX in patients who have developed M-pneu has led to recurrence of lung injury and in many cases to death (11, 49). There are single cases, however, in which the drug has been re-introduced successfully (50).

### Rheumatoid Arthritis Related Interstitial Lung Disease

RA is not merely a disease of the joints. It is a true systemic inflammatory disease with effects on many organs and organ systems. A variety of pulmonary manifestations can be seen in RA including pulmonary nodules, pleural effusions, bronchiectasis, and, most importantly, ILD (2).

#### Epidemiology

ILD is a frequently under-recognized complication of RA. The estimated prevalence is heavily dependent on the ascertainment method used. Bongartz et al. reported a lifetime risk of 7.7%, a 9-fold increase over the general population (51). Studies using the ERAS and ERAN early arthritis cohorts as well as the ILD specific BRILL study in the UK reported a prevalence of RA-ILD of 3–5% (52, 53) (Table 2). All of these studies identified clinical RA-ILD; if screening of asymptomatic individuals with RA is utilized, the prevalence of ILD increases depending on the performance characteristics of the screening methodology used. High resolution CT scanning identifies ILD in 19–67% of RA patients depending on the thresholds for diagnosis employed (54, 55). A study performing unselected histological assessment of pulmonary tissue in RA patients revealed evidence of ILD in 80% of patients (69). For these studies in which ILD was diagnosed based on radiologic and histological data, it should be noted that they probably overestimate clinically relevant RA-ILD. Patients were included irrespective of pulmonary symptoms and many of them had normal PFTs.

**Table 2:** Comparison of clinical and imaging features in M-pneu *vs*. RA-ILD.

	**MTX-pneu**	**RA-ILD**	**References**
Frequency in RA	0.3–11.6%	3–5% (clinical diagnosis) 19–67% (radiological diagnosis)	(3–6, 52–55)
Course	Usually acute or sub-acute, within the first year of treatment	Usually chronic^*^	(8, 10, 38)
Clinical symptoms	Fever, dry cough, dyspnoea	Fever, dry cough, dyspnoea	(3, 11, 12)
Imaging findings	Mostly NSIP No specific predilection New or evolving diffuse interstitial or mixed interstitial and alveolar infiltrates Diffuse and patchy bilateral ground glass opacity with or without reticulation Cellular interstitial infiltrates, granulomas, diffuse alveolar damage	UIP > NSIP Basal and peripheral distribution CXR: punctate and reticulonodular densities and coarse reticulations CT: basal cystic changes (honeycombing, periperheal reticular opacities, bronchioloectasis Lower lobe volume loss in the course of disease	(45–47, 56–58)
Bronchoalveolar lavage	Lymphocytic more common that neutrophilic pattern	Neutrophilic or lymphocytic pattern^#^	(36, 40, 41, 59)
Histopathology	Interstitial infiltrates by lymphocytes, histiocytes and eosinophils sometimes with non-caseating granulomas	UIP, NSIP > OP and other patterns	(12, 38, 47, 53, 60)
Treatment options	Discontinuation of MTX Glucocorticoids Rarely cyclophosphamide, TCZ	Glucocorticoids MTX or LEF possibly beneficial anti-TNF, ATC, TCZ: inconclusive data rituximab: possibly beneficial	(3, 7, 8, 12, 48, 61–68)

*ATC, abatacept; CT; computed tomography; CXR, chest x-ray; LEF, leflunomide; MTX, methotrexate; NSIP, non-specific interstitial pneumonia; OP, organizing pneumonia; TCZ, tocilizumab; TNF, tumor necrosis factor; UIP, usual interstitial pneumonia*.

* *Cases of fulminant RA-ILD have been described*.

# *It has been suggested that RA patients with clinical/radiologic findings of lung involvement have neutrophilic pattern and those without a lymphocytic pattern*.

#### Pathogenesis and Risk Factors

Increasing evidence supports a primary role for the lung in initiating RA pathogenesis and RA-ILD may occur prior to the onset of the joint disease (70–72). Known predictors of RA-ILD include RA severity, age, male sex, smoking, and seropositivity for rheumatoid factor or anti-citrullinated peptide antibodies anti-citrullinated peptide antibodies (51, 71) (Table 1). In the past several years, biomarkers for RA-ILD have been suggested: Citrullinated isoforms of heat shock protein 90 (hsp90) have been shown to be potentially useful as a biomarker of RA-ILD (73). Hsp90 could also be identified in BAL specimens (74). Recently, the gain-of-function MUC5B promoter variant rs35705950, has been found to be associated with the development of ILD in RA patients with an Odds Ratio of 3.1 (75). This is especially interesting given that the same MUC5B variant is the strongest known risk factor for idiopathic pulmonary fibrosis (IPF), which shares many similarities with RA-ILD (76).

#### Clinical Symptomatology and Laboratory Findings

The clinical findings in RA-ILD are similar to those previously described for M-pneu with dyspnoea and non-productive cough with or without fever predominating (7). Most typically, RA-ILD develops insidiously over time and may be present and asymptomatic for a significant period. This diagnostic delay may be further exacerbated by the fact that patient's rheumatoid joint disease may limit their ability to exercise sufficiently to precipitate exertional dyspnea. Clinical examination findings may be absent in early disease but ultimately the majority of patients will have fine bibasal crepitations (77). The majority of those with an usual interstitial pneumonia (UIP) pattern RA-ILD will also develop clubbing, similar to IPF patients (77). Radiologic findings are of little help in distinguishing the two disorders with a significant degree of overlapping features (46). However, a key distinguishing feature can be chronicity. MTX-pneu is typically a fulminant acute process (11) (Table 2). A more indolent subacute or chronic development of radiologic findings strongly favors RA-ILD. In this scenario, historic radiologic imaging demonstrating evidence of similar but early ILD changes argues against MTX-pneu. However, RA-ILD may present as a fulminant and potentially fatal process, including early in the disease process (2, 78, 79).

#### Diagnosis

The diagnosis of RA-ILD can generally be made by a combination of clinical features as described above and congruent findings on chest imaging. It is important to remember that RA patients are, at least equally, and in often cases more likely, to develop other causes of dyspnea and cough than the general population. For example, the risk of infection, including atypical infections, pulmonary emboli, and lung cancer, are all increased in RA patients (80–82). PFTs may provide evidence of restrictive lung disease with a reduced TLCO/DCLO generally being the first manifestation.

Bronchoscopy and BAL may be performed to rule out other diagnoses. BAL is frequently abnormal in RA-ILD, but the findings are non-specific and rarely diagnostically useful. In rare cases open lung biopsy may be needed to confirm a diagnosis, in general when an alternative diagnosis is suspected.

#### Imaging Features

**Figure 2:**
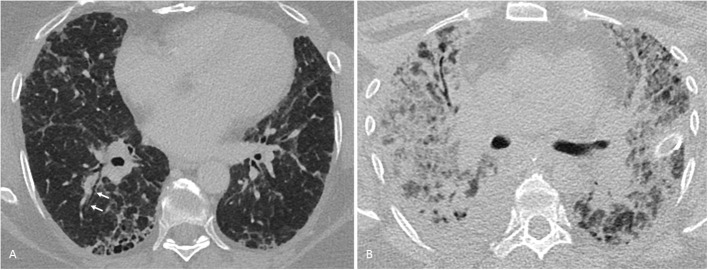
Interstitial lung disease in a 56-year-old woman with rheumatoid arthritis. **(A)** One millimeter transverse axial CT-section through the lung bases show subpleural honeycombing and early traction bronchiectasis (arrows), consistent with a usual interstitial pneumonia pattern. **(B)** Nine months later, the patient developed severe dyspnea at rest and required mechanical ventilation. On bronchoalveolar lavage, influenza A virus was found to be present. A follow-up CT now showed a small right-sided pleural effusion and multifocally confluent consolidation, partially obscuring equally patchy bilateral ground-glass opacification. A few thickened septae (crazy-paving pattern) could be delineated (not shown). These findings were consistent with a viral pneumonia. Despite extracorporeal membrane oxygenation therapy, the patient deceased.

Apart from treatment-related complications, the thoracic manifestations of RA are plentiful (56) and include pleural changes, large airway involvement and, more so than with other collagen vascular diseases, a usual interstitial pneumonia (UIP) pattern of interstitial lung disease as distinct from a non-specific interstitial pneumonia (NSIP) or other patterns (71, 83) (Table 2). Clinically relevant ILD is less common, comprising basal cystic changes (honey combing), peripheral reticular opacities and bronchioloectasis, best seen on CT scanning, and lower lobe volume loss which may advance in the chronic stage (Figure 2). Bronchiolitis obliterans has been described in RA, while follicular bronchiolitis is more common, showing small nodular changes on CT. Rheumatoid nodules as large as 5 cm are more likely in men, typically occur in smokers and may be seen prior to the articular manifestation of the disease. Nodules may cavitate, occasionally calcify and rarely rupture.

#### Histologic Findings

Findings on BAL are generally abnormal but non-specific in RA-ILD. Common findings include some form of neutrophil or lymphocytic predominant leucocytosis, or alterations in T-lymphocyte ratios (36, 41, 59, 84–86). Histologic findings are congruent with those seen with the underlying ILD phenotype, including neutrophilic or lymphocytic infiltrates, and fibrotic changes. A number of histopathological findings have been suggested to aid in the differentiation of MTX-pneu from RA-ILD including type II pneumocyte hyperplasia and fibroblast proliferation (11). However, these features have also been reported in RA-ILD.

#### Treatment

Glucocorticoids remain an important part of the acute management of RA-ILD. The optimum longer-term management of RA-ILD is uncertain, however, given the known factors predictive of RA-ILD described above it is logical that good RA disease control should be the cornerstone of any strategy (61). This is supported by the significant decline in the reported frequency of RA-ILD as RA treatment options have advanced (87). Given its proven efficacy in RA joint disease there is good reason to expect that MTX may be a justified part of any treatment strategy in an RA patient with ILD; evidence to support this strategy is beginning to emerge (60, 88). Despite previous concerns over potential pulmonary toxicity with leflunomide, this agent also appears to be potentially beneficial for RA-ILD (62). In the setting of RA-ILD, the choice of biological therapy is not clear: A recent review of the literature identified seven studies and 28 case reports, which showed an increased mortality with the use of tumor necrosis factor-inhibitors (TNF-i) (63). In this analysis, female sex and longer disease duration were associated with ILD onset or worsening (63). The heterogeneity in the reported outcome measures was too large to draw any firm conclusions. Other agents, such as Abatacept (ATC) have been investigated in few studies: In a Japanese study, deterioration of RA-ILD was described in 11 of 131 patients (8.4%) and was associated with concomitant MTX use (Odds Ratio of 12.75) (89). By contrast, a multicentric analysis from Spain concluded that ATC was associated with stable ILD in about two thirds of the patients (64). The role of TCZ in RA-ILD is less clear. A retrospective study in Japan showed worsening of ILD with TCZ in only six of 78 patients (7.7%) (65) or even improvement (38). These findings are in line with data from clinical trials in Systemic sclerosis (90), where it has been shown to preserve lung function, although this was not the primary endpoint.

Preliminary evidence of a particular role for Rituximab (RTX) is beginning to emerge (66, 67, 91, 92). An observational study of 56 patients with RA-ILD treated with RTX showed that 16% improved and 52% remained stable; a particularly impressive response given the aggressive natural history of RA-ILD (11, 66). This is logical given the association of RA-ILD with other known predictors of Rituximab response, in particular seropositivity (68).

**Figure 3:**
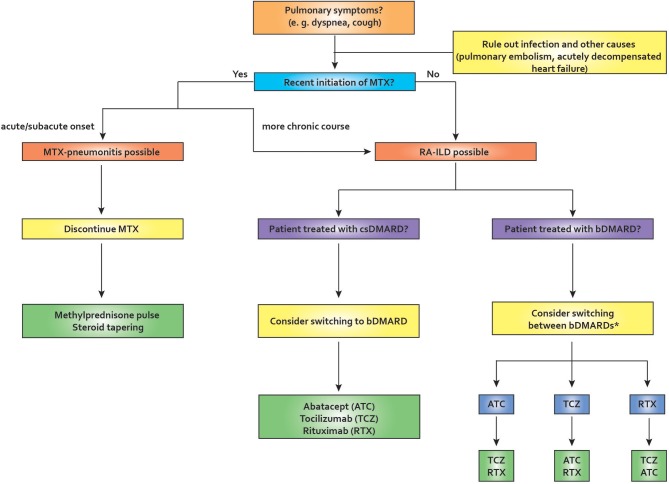
Proposed algorithm for pulmonary symptoms in rheumatoid arthritis. In the setting of recent MTX initiation, MTX-pneu is always a concern, especially if the onset of symptoms is acute or sub-acute. In this case, MTX needs to be stopped and usually glucocorticoid therapy and supportive care in an intensive care unit is required. If the onset is more insidious, RA-ILD is a possibility. After ruling out other causes of pulmonary symptoms, management should depend on various factors, including comorbities, age, disease activity, and others. If a patient is diagnosed as having RA-ILD and receives a csDMARD, switching to a bDMARD may be appropriate. If a patient is already on bDMARD therapy, switching therapies may be required. Many authors tend to avoid TNF-inhibitors in this situation, but the evidence is weak. ATC, abatacept; bDMARD, biological disease-modifying antirheumatic drug; csDMARD, conventional synthetic disease-modifying antirheumatic drug; ILD, interstitial lung disease; MTX, methotrexate; MTX-pneu, MTX-pneumonitis; RA, rheumatoid arthritis; RTX, rituximab; TCZ, tocilizumab. *TNF-inhibitors have been reported to be associated with worsening lung function in RA-ILD (weak evidence level).

Other agents are currently under investigation in the treatment of RA-ILD: The anti-fibrotic tyrosine kinase inhibitor nintedanib has been shown to be effective in an animal model of RA-ILD; the same agent has demonstrable efficacy in RCTs in IPF and, recently, also in systemic sclerosis (93–95). Another anti-fibrotic agent, pirfenidone, has been shown to downregulate profibrotic pathways in a bleomycin-induced mouse model and lung biopsy specimens from RA-ILD patients (96). Figure 3 depicts our proposed treatment approach to the treatment of pulmonary manifestations in RA.

#### Prognosis

ILD in general has a poor prognosis, however, this is even more true of RA-ILD, which has an ominous prognosis with a Hazard Ratio (HR) for death of 2.86 (51). Overall, respiratory causes are the second most common cause of death in patients with RA; symptomatic RA-ILD contributes 13% of the excess mortality associated with RA (51, 53, 97). Median survival following a diagnosis of RA-ILD is <3 years (2, 97). Acute fulminant RA-ILD occurring rapidly following disease onset is well-documented and frequently fatal (2, 78, 79). RA-ILD patients with a UIP pattern on imaging have increased mortality compared to other patterns, with a relative risk of 2.39 for UIP compared to NSIP (98). As well as the inherent mortality associated with RA-ILD itself, these patients are also at significantly increased risk of pulmonary infection (71, Figure 2).

## Conclusions and Future Research Directions

Methotrexate pneumonitis usually presents acutely but its incidence has been decreasing over time. Suspension of MTX and administration of glucocorticoid pulse therapy are usually required. In the long term, MTX therapy may associate with a lower incidence of RA-ILD, thus questioning the fear of progressive pulmonary fibrosis associated with this agent. Regarding bDMARDs, ATC, TCZ, or RTX appear more promising than TNF-i in patients requiring more intense immunosuppression although the evidence base for this remains weak.

Future studies should aim at determining the exact prevalence of RA-ILD in early stage RA patients and will certainly rely on PFTs and imaging with CT at baseline and during the disease course to help identify patients at high risk for progression.

## Author Contributions

GF, EN, and RC wrote the first draft of the manuscript. PK edited and revised the manuscript and drafted the figures. JL edited the manuscript and contributed figures. All authors revised the manuscript critically and approved the final version of the manuscript.

### Conflict of Interest

The authors declare that the research was conducted in the absence of any commercial or financial relationships that could be construed as a potential conflict of interest.
